# Prevalence and Patterns of Nitrosatable Drug Use among U.S. Women during Early Pregnancy

**DOI:** 10.1002/bdra.20808

**Published:** 2011-04-06

**Authors:** Jean D Brender, Katherine E Kelley, Martha M Werler, Peter H Langlois, Lucina Suarez, Mark A Canfield, the National Birth Defects Prevention Study

**Affiliations:** 1 Texas A&M Health Science Center, School of Rural Public Health, College StationTexas; 2 Slone Epidemiology Center at Boston UniversityBoston, Masachusetts; 3 Birth Defects Epidemiology and Surveillance Branch, Texas Department of State Health ServicesAustin, Texas; 4 Environmental Epidemiology and Disease Registries, Texas Department of State Health ServicesAustin, Texas

**Keywords:** nitrosatable drugs, pregnancy, *N*-nitroso compounds, nitrosation, secondary amine, tertiary amine

## Abstract

**BACKROUND:**

Experimental evidence indicates that certain drugs, that are secondary or tertiary amines or amides, form *N*-nitroso compounds in the presence of nitrite in an acidic environment. Nitrosatable drugs have been associated with birth defects in a few epidemiologic studies. This study describes the prevalence and patterns of nitrosatable drug use among U.S. women during early pregnancy and examines maternal factors associated with such use.

**METHODS:**

Data were analyzed from the National Birth Defects Prevention Study and included 6807 mothers who gave birth to babies without major congenital malformations during 1997 to 2005. Information was collected by telephone interview about medication use, demographic factors, and maternal health. Drugs taken during the first trimester were classified according to nitrosatability, amine and amide functional groups, and primary indication of use.

**RESULTS:**

Approximately 24% of the women took one or more nitrosatable drugs during the first trimester, including 12.4%, 12.2%, and 7.6% who respectively took secondary amines, tertiary amines, or amides. Five of the ten most commonly taken drugs were available over the counter. Women who were non-Hispanic white (29.5%), with 1 year or more college education (27.3%) or 40 years or older (28.8%) had the highest prevalence of use. Supplemental vitamin C, an inhibitor of nitrosation, was not taken by 41.6% and 19.3% of nitrosatable drug users during the first and second months of pregnancy, respectively.

**CONCLUSIONS:**

In this U.S. population, ingestion of drugs classified as nitrosatable was common during the first trimester of pregnancy, especially among non-Hispanic white, more educated, and older mothers. Birth Defects Research (Part A) 2011. © 2011 Wiley-Liss, Inc.

## INTRODUCTION

Extensive experimental evidence indicates that *N*-nitroso compounds can be formed in vivo via the reaction of nitrosatable amines or amides and nitrosating agents such as nitrite in an acidic environment, as is found in the stomach (Preussmann,^1984^). A variety of drugs have been classified as contributing amines (secondary or tertiary) or amides in the endogenous formation of *N*-nitroso compounds (Lijinsky,^1974^). Supporting evidence for these classifications comes from standardized experiments of specific compounds using the World Health Organization Nitrosation Assay Procedure (Gillatt et al.,^1984^; Brambilla et al.,^1985^) or simulated human gastric conditions (Ziebarth and Teichmann,^1980^; Gillatt et al.,^1984^; Sakai et al.,^1984^; Gillatt et al.,^1985^; Ohta et al.,^1986^; Ziebarth et al.,^1989^) that resulted in the production and identification of nitrosamines and nitrosamides. In these tests, drugs containing secondary amine or amide groups had greater yields of *N*-nitroso compounds than did those containing tertiary amines; however, nitrosation of tertiary amines and amides resulted in the production of known carcinogens (e.g., n-nitrosodimethylamine, nitrosoureas). In addition, drugs have been classified as theoretically nitrosatable through evaluation of their chemical structure (McKean-Cowdin et al.,^2003^), or through genotoxic testing of nitrosation products, with and without identification of specific *N*-nitroso compound (Andrews et al.,^1980^; Alba et al.,^1988^; Ozhan and Alpertunga,^2003^).

In a series of experiments on mice, Platzek et al. (1983) noted that acetoxymethyl-methylnitrosamine, which has the same active intermediate metabolite as n-nitrosodimethylamine, increased exencephaly, cleft palate, limb malformations, and other skeletal anomalies at doses not associated with maternal toxicity. It is noteworthy that a sizable number of nitrosatable drugs form n-nitrosodimethylamine in the presence of nitrite (McKean-Cowdin et al.,^2003^; Brambilla and Martelli,^2007^).

To date, five epidemiologic studies have examined the relation between maternal exposure to nitrosatable drugs and birth defects in offspring. Using data from the Collaborative Perinatal Project, Olshan and Faustman (1989) identified a total of 13 nitrosatable drugs taken during the first 4 months of pregnancy. Relative to women not taking these drugs, women who took one or more nitrosatable drugs were more likely to have infants with eye, musculoskeletal, or gastrointestinal malformations; hydrocephaly; craniosynostosis; or spina bifida. Findings of two subsequent studies (Gardner et al.,^1998^; Kallen and Robert-Gnansia,^2005^) indicated positive associations between prenatal exposure to three of the drugs identified by Olshan and Faustman (1989) as nitrosatable (chlordiazepoxide, nitrofurantoin, and chlorpheniramine) and craniosynostosis. In a study of nitrates and neural tube defects, Croen et al. (2001) also used the Olshan and Faustman list to classify women's exposure to nitrosatable drugs and found no association between taking such drugs and neural tube defects. However, Brender et al. (2004) noted a significant positive association between reported use of drugs with potential for nitrosation and neural tube defects in offspring among Mexican American women who also had a higher dietary intake of nitrites.

Results of two epidemiologic studies have also suggested an association between prenatal exposure to nitrosatable drugs and childhood cancer (Preston-Martin et al.,^1982^; Olshan and Faustman,^1989^), although four subsequent studies found no association between these drug exposures and childhood brain tumors (Carozza et al.,^1995^; McKean-Cowdin et al.,^2003^; Cardy et al.,^2006^) or neuroblastoma (Cook et al.,^2004^).

Exposure measurement was a limitation in previous epidemiologic studies of prenatal exposures of nitrosatable drugs. First, study participants were often asked to recall drug use in pregnancies that occurred up to 20 years earlier in the childhood cancer studies (Preston-Martin et al.,^1982^; Carozza et al.,^1995^; McKean-Cowdin et al.,^2003^; Cook et al.,^2004^; Cardy et al.,^2006^). Second, classifications of drugs as nitrosatable were incomplete (Preston-Martin et al.,^1982^; Olshan and Faustman,^1989^; Carozza et al.,^1995^; Gardner et al.,^1998^; Brender et al.,^2004^). Brambilla and Martelli (2007) identified 182 drugs that had been tested, of which 173 (95%) were found to form *N-*nitroso compounds or other reactive species, and they acknowledged the possibility that additional published and unpublished results could extend this list. Only one of the previous birth defect studies classified nitrosatable drugs by indication of use, and none classified these drugs by molecular structure (i.e. secondary amines, tertiary amines, or amides). Research has also indicated that certain compounds can inhibit endogenous nitrosation, with ascorbic acid being the most thoroughly studied (Mirvish,^1994^). Brambilla and Martelli (2007) noted that the formation of *N*-nitroso compounds from drug-nitrite interactions could be reduced by taking ascorbic acid with nitrosatable drugs. Previous studies have not reported prevalence of vitamin C supplementation among women who took drugs classified as nitrosatable or indicated the effects of vitamin C intake on any associations between nitrosatable drug use and adverse pregnancy outcomes. Accounting for vitamin C use when assessing the effects of nitrosatable drugs on pregnancy outcomes may prove to be critically important. The purpose of our study was (1) to describe the prevalence and patterns of nitrosatable drug use among U.S. women during the first trimester of pregnancy, including type of drug (secondary or tertiary amine, amide) and indication of use; (2) to examine maternal factors associated with nitrosatable drug use; and (3) to investigate the prevalence of supplemental vitamin C use among women who took nitrosatable drugs in early pregnancy.

## MATERIALS AND METHODS

### Study Population

To assess the prevalence, patterns of use, and factors related to nitrosatable drug use among childbearing women, we used data from the National Birth Defects Prevention Study (NBDPS), an ongoing population-based case-control study of birth defects in the United States that began in 1997. Ten Centers for Birth Defects Research and Prevention (CBDRP; Arkansas, California, Georgia, Iowa, Massachusetts, New Jersey [1998 to 2002], New York, North Carolina [2003 to present], Texas, and Utah [2003 to present]) participate in this national study. Mothers of cases and controls were invited to participate within 24 months after the expected due date of the index pregnancy. Information about the study populations and methods used in the NBDPS has been described in detail elsewhere (Yoon et al.,^2001^). The institutional review boards in each state and the Centers for Disease Control and Prevention approved the study protocol, and the Texas A&M Institutional Review Board also approved the project from which the present analysis is a substudy.

Analyses for our study included control women only, whose pregnancy due dates were between October 1, 1997, and December 31, 2005. Control-infants were live births without major birth defects who were residents of the geographic areas covered by one of the CBDRP population registries at the time of delivery. These controls were selected randomly from either live birth certificates (Iowa, Massachusetts, New Jersey, Metropolitan Atlanta, North Carolina, and Utah) or hospital records (New York, California, Arkansas, and Texas) (Centers for Disease Control and Prevention, 2005). For those states that selected controls from hospitals, a systematic random sampling scheme was used that selected infants in proportion to the number of births in each hospital in a given geographic area (Yoon et al.,^2001^). Control-infants were not eligible if they had a major birth defect, were not a resident of one of the geographic areas covered by CBDRP, were adopted or in foster care, had a deceased mother, or were stillborn (The National Birth Defects Prevention Study Prototcol, Centers for Disease Control and Prevention, 2005).

### Data Collection

As part of the NBDPS, women were interviewed by telephone by trained interviewers with a standard questionnaire after oral informed consent was obtained. The interview lasted for approximately 1 hour and covered topics regarding maternal health (diseases and illnesses, fever, injuries, medications), pregnancy issues (pregnancy history, prenatal care, morning sickness); diet or substance abuse (vitamins, food supplements, dietary assessment, caffeine consumption, tobacco, alcohol, street drugs), demographic characteristics, and occupational and environmental exposures (Yoon et al.,^2001^). Women were questioned about drugs taken (start and stop dates, frequency of use) for specific illnesses and diseases (e.g., asthma, diabetes, high blood pressure, infections, respiratory illnesses, seizures) and about specific products (e.g., ampicillin, metoprolol, propranolol, phenytoin, pseudoephedrine). From these interviews, reported products were linked to their active ingredients by the Slone Epidemiology Center Drug Dictionary (Kelley et al.,^2003^). Women were also questioned about vitamin use, including prenatal vitamins, multivitamins, and single vitamins. A total of 6807 (66.2%) of eligible control mothers (expected delivery dates between October 1, 1997, and December 31, 2005) participated in the interview.

### Classification of Nitrosatable Drugs

All orally administered prescription and nonprescription medications reported by control women and their active ingredients were first identified. Orally inhaled medications (e.g., albuterol, epinephrine) were included because they may be deposited in the mouth and throat, mixed with saliva, and swallowed. Products applied topically to the mucous membranes of the eye and nose were excluded. The resulting group of active ingredients was cross-referenced against previously compiled lists of nitrosatable medicinal compounds (McKean-Cowdin et al.,^2003^; Brambilla and Martelli,^2007^) and categorized based on the presence of amine (primary, secondary, tertiary) and amide functional groups in their chemical structure. Primary amines do not form stable *N*-nitroso compounds and were excluded. A number of compounds were available for inclusion in multiple categories (e.g., atenolol is both a secondary amine and amide). The structures of all remaining active ingredients were evaluated for the presence of amine and amide functional groups and were checked for any additional published evidence of nitrosatabilty using Medline and Internet sources. Finally, each component was categorized by its primary indication or therapeutic use (e.g., analgesic, antiinfective) and pharmacologic class (e.g., opioid, macrolide; Supplemental Table 1).

A dataset for each nitrosatable component was created with the NBDPS Data Analysis Tools and Database Version 7.04, Medication Exposure Data Set Creation System, that included variables for whether the drug was taken during the first trimester and the number of days taken during that period. Individual drug components were grouped into their respective nitrosatable groups (secondary amines, tertiary amines, amides) and indication groups (e.g., analgesic, antihistamine, stimulant).

### Data Analysis

Prevalence of intake by nitrosatable category, therapeutic use, and individual components was calculated by dividing the number of NBDPS participants who reported taking the respective components during the first trimester of pregnancy by the total number of women for which use of that component or groups of components was known (excluding unknowns). We also calculated prevalence of nitrosatable use by various maternal characteristics, including maternal race or ethnicity (non-Hispanic white, non-Hispanic black, Hispanic, Asian, Pacific Islander, other); age at delivery (seven age groups), and years of education (0 to 8 years, 9 to 11 years, 12 years, 13 to 15 years, >15 years). Prevalence of use by state of residence was adjusted by maternal race or ethnicity using direct adjustment procedures (Szklo and Nieto,^2007^) and the racial or ethnic distribution of all NBDPS comparison mothers combined as the standard population. Secular trends of nitrosatable drug use were examined for expected dates of delivery from 1997 to 2005. Although we focused on nitrosatable drug use during the first trimester, we also examined the prevalence of these drug exposures during the periconceptional period (1 month before to 1 month after conception), the exposure window that would be more relevant for some malformations such as neural tube defects. Because vitamin C supplement use varied considerably by month during the first trimester, we examined prevalence of supplement use (none, less than daily, daily) separately during the first, second, and third months of pregnancy by nitrosatable drug use during the first trimester.

## RESULTS

A total of 1572 (23.6%) of women took one or more nitrosatable drugs during the first trimester of pregnancy. Approximately 12% of women reported taking drugs that were secondary or tertiary amines, with 7.6% reporting use of drugs classified as nitrosatable amides. The most common indications of use varied by drug category, with decongestants, asthma medications, and antidepressants being the most common among secondary amines. Antihistamines, antiemetics, and cough suppressants were the most common tertiary amines, and antiinfectives and stimulants (e.g., caffeine) were the most common amide drugs (Table 1). Although rank varied by category, the most common nitrosatable components included pseudoephedrine (7.7% of the women), amoxicillin (4.0%), promethazine (3.1%), albuterol (2.7%), dextromethorphan (2.1%), diphenhydramine (1.7%), chlorpheniramine (1.6%), doxylamine (1.2%), caffeine (1.0%), and fluoxetine (0.8%; Table 2).

**Table 1:** Prevalence of Nitrosatable Drug Use by Indication during the First Trimester of Pregnancy, National Birth Defects Prevention Study Control Women, 1997–2005

Nitrosatable Drug Components^a^	Number	%
**Any nitrosatable drug**	1572	23.6
**Any secondary amine**^b^	828	12.4
Asthma medications: beta adrenergic	182	2.7
Antidepressant medications	92	1.4
Selective serotonin reuptake inhibitor	88	1.3
Tricyclic	4	0.1
Cardiovascular medications	32	0.5
Beta blocker	23	0.3
Thiazide diuretic	6	0.1
Decongestant	518	7.8
Antidiabetic, biguanide^c^	16	0.2
Gastrointestinal, H2 blocker	30	0.4
Muscle relaxant, migraine	7	0.1
**Any tertiary amine**^b^	820	12.2
Analgesic	91	1.4
Opoids	86	1.3
Anticholinergic	14	0.2
Antidepressant, Tricyclic	5	0.1
Antidiabetic, Biguanide^c^	16	0.2
Antidiarrheal, Opoid	1	0.0
Antiemetic-prokinetic	253	3.8
Antihistamine	12	0.2
Phenothiazine	224	3.3
Prokinetic^d^	25	0.4
Antiepileptic^e^	10	0.1
Antihistamine	336	5.0
Antiinfective	38	0.6
Macrolide	27	0.4
Tetracylines	3	0.0
Benzodiazepine	8	0.1
Cardiovascular	9	0.1
Beta blocker	1	0.0
Calcium channel blocker	3	0.0
Antihypertensives^f^	5	0.1
Cough suppressant	139	2.1
Gastrointestinal, H2 blocker	30	0.4
Migraine	6	0.1
Nicotine replacement	1	0.0
Stimulant^g^	64	1.0
**Any amide**^b^	512	7.6
Antiemetic-prokinetic^d^	37	0.6
Antiepileptic	16	0.2
Antiinfective	375	5.6
B-Lactam	334	5.0
Macrolide	3	0.0
Sulfonamide^h^	38	0.6
Tetracyclines	3	0.0
Benzodiazepine	10	0.1
Cardiovascular	17	0.3
Beta-blocker	11	0.2
Calcium channel blocker	1	0.0
Diuretic	6	0.1
Migraine	6	0.1
Stimulant^g^	64	1.0

a Complete information available for 6649 women for any nitrosatable drugs, 6678 women for any secondary amines, 6708 women for any tertiary amines, and 6701 women for any amides.

b See Supplemental Table 1 for a listing of nitrosatable drugs.

c Metformin.

d Metoclopramide.

e Carbamazepine.

f Hydralazine, clonidine.

g Caffeine.

h Sulfamethoxazole.

**Table 2:** Most Commonly Used Nitrosatable Drug Components during the First Trimester of Pregnancy, National Birth Defects Prevention Study Control Women, 1997–2005

Nitrosatable Drug Components^a^	Number	%
Any secondary amine (24 components)^b^	828	12.4
Albuterol	178	2.7
Fluoxetine	53	0.8
Paroxetine	36	0.5
Pseudoephedrine	515	7.7
Ranitidine	29	0.4
Any tertiary amine (51 components)^b^	820	12.2
Chlorpheniramine	104	1.6
Dextromethorphan	139	2.1
Diphenhydramine	115	1.7
Doxylamine	82	1.2
Promethiazine	207	3.1
Any amide (22 components)^b^	512	7.6
Amoxicillin	271	4.0
Caffeine	64	1.0
Cephalexin	44	0.7
Metoclopramide	25	0.4
Sulfamethoxazole	38	0.6

a Complete information available for 6649 women for any nitrosatable drugs, 6678 women for any secondary amines, 6708 women for any tertiary amines, and 6701 women for any amides.

b The five most common components listed under each class of nitrosatable drugs.

**Figure 1:**
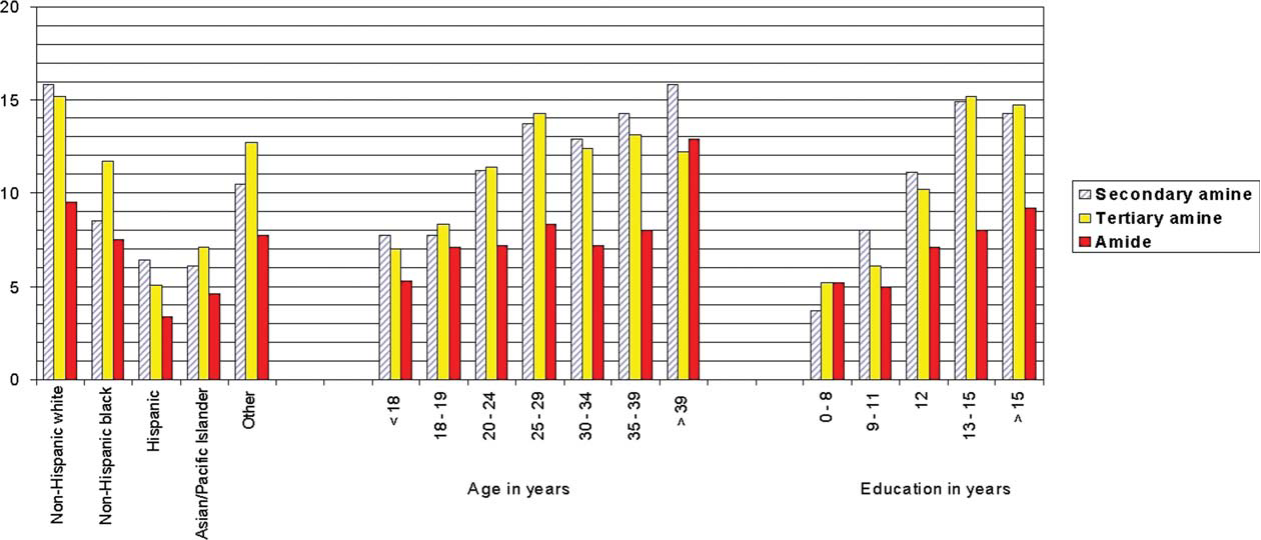
Prevalence (%) of nitrosatable drug use during the first trimester by maternal characteristics, National Birth Defects Prevention control women, 1997–2005. [Color figure can be viewed in the online issue, which is available at wileyonlinelibrary.com.]

During the first trimester, the prevalence of nitrosatable drug use varied by maternal race or ethnicity, age, and education in this study population (Fig. 1). Non-Hispanic white women had the highest prevalence of nitrosatable drug use during this period (29.5%) as well as for the sub-categories of secondary amines (15.8%), tertiary amines (15.2%), and amides (9.5%). Hispanic women reported the lowest use (11.6%) of nitrosatable drugs. Prevalence of use of these drugs generally increased with maternal age, although less of an age trend was noted with nitrosatable amide drugs. Women who had 1 year or more of college were more than twofold as likely (27.3%) than women with less than 9 years of education (10.7%) to use any nitrosatable drugs. This educational pattern was stronger for secondary and tertiary amines than for amides.

**Figure 2:**
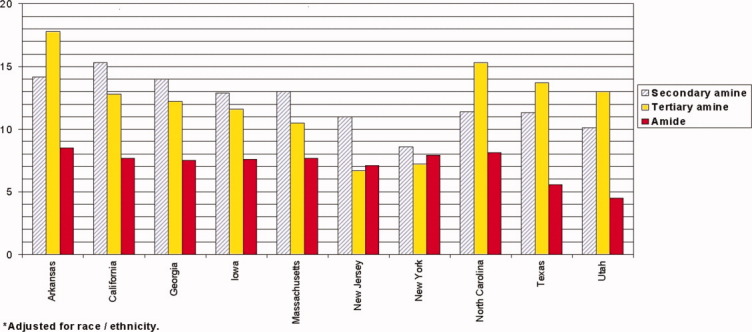
Prevalence (%) of nitrosatable drug use during the first trimester by state of residence, National Birth Defects Prevention Study control women, 1997–2005. [Color figure can be viewed in the online issue, which is available at wileyonlinelibrary.com.]

Adjustment for maternal race or ethnicity reduced prevalence differences of nitrosatable drug use by state of residence, although some variation still remained by geographical area (Fig. 2). NBDPS control mothers who resided in Arkansas had the highest prevalence of nitrosatable drug use overall (28.0%), for tertiary amines (17.8%) and amides (8.5%), but California participants had the highest prevalence of secondary amine use (15.3%).

For the period of 1997 to 2005, prevalence of any nitrosatable drug use varied only minimally by year, ranging from 22.4% in 2001 to 24.5% in 2004 (data not shown). No overall secular trends were noted for secondary and tertiary amines or amides.

During the periconceptional period, 16.8% of women reported taking one or more nitrosatable drugs, and approximately 10%, 9%, and 5% reported taking drugs classified as secondary amines, tertiary amines, or amides, respectively. Similar to patterns noted for the first trimester, women who took nitrosatable drugs during the periconceptional period were most likely to be non-Hispanic white (21.5%), have 1 year or more of college education (19.4%), or be 40 years or older (22.1%).

Among women who took nitrosatable drugs, <40% reported daily use of a supplement with vitamin C during the first month of pregnancy (Table 3), although approximately 80% took such a supplement daily by the third month. In the first month of pregnancy, between 40 and 41% (depending on the type of nitrosatable drug taken) of participants who took nitrosatable drugs did not take any supplements with vitamin C; the proportion of nonusers dropped to 12.3% by the third month of pregnancy.

**Table 3:** Vitamin C Supplementation^a^ among Nitrosatable Drug Users during the First Three Months of Pregnancy, National Birth Defects Prevention Study Control Women, 1997–2005

	Number (%) who took supplement with vitamin C in the first month postconception			Number (%) who took supplement with vitamin C in the second month postconception			Number (%) who took supplement with vitamin C in the third month postconception		
			
Nitrosatable drug taken	None	Less than daily	Daily	None	Less than daily	Daily	None	Less than daily	Daily
Any	652 (41.6)	311 (19.8)	604 (38.5)	303 (19.3)	299 (19.1)	965 (61.6)	193 (12.3)	125 (8.0)	1249 (79.7)
Secondary amine	334 (40.4)	165 (20.0)	327 (39.6)	158 (19.1)	149 (18.0)	519 (62.8)	89 (10.8)	71 (8.6)	666 (80.6)
Tertiary amine	333 (40.8)	175 (21.4)	308 (37.7)	139 (17.0)	176 (21.6)	501 (61.4)	98 (12.0)	67 (8.2)	651 (79.8)
Amide	205 (40.0)	107 (20.9)	200 (39.1)	93 (18.2)	97 (18.9)	322 (62.9)	58 (11.3)	37 (7.2)	417 (81.4)

a Supplementation from single, prenatal, or multi-vitamin preparations.

## DISCUSSION

In this population-based study of U.S. women who gave birth to infants without major birth defects, use of nitrosatable drugs was fairly common during the first trimester of pregnancy. Nearly one quarter of the women took such drugs early in pregnancy with an even higher prevalence of exposure among non-Hispanic white women, women over 40 years of age, and women with 13 years or more of education. The prevalence of nitrosatable drug use was lower during the periconceptional period than for the first trimester, which might be expected because of the shorter period (2 vs. 3 months). Regarding maternal race or ethnicity, age, and education, similar patterns of nitrosatable drug usage were noted during these periods. Pseudoephedrine, a nitrosatable secondary amine that is available without a prescription, was the most common nitrosatable drug in this population. It is also noteworthy that four of the five most commonly taken drugs classified as nitrosatable tertiary amines are also available over the counter. During the first and second months of pregnancy, a sizable proportion of women who took nitrosatable drugs denied taking vitamin supplements with vitamin C (41.6% and 19.3%, respectively), a well-documented inhibitor of nitrosation. By the third month of pregnancy, approximately 80% reported taking supplements that most likely contained vitamin C.

Compared with prevalence estimates in previously published studies, the present study has the highest reported prevalence of nitrosatable drug use during early pregnancy. Olshan and Faustman (1989) published one of the first studies on nitrosatable drug use during pregnancy. Among 51,228 pregnancies followed between 1959 and 1965 by the U.S. National Collaborative Perinatal Project, 3.5% of mothers used nitrosatable drugs during the first 4 months of pregnancy, and 11.8% took such drugs anytime during pregnancy. Users in that study were more likely to be white, have more education, and be slightly older than nonusers, which is consistent with the trends noted in the present study. The investigators identified a total of 13 drug components deemed to have nitrosation potential, far fewer than those identified in the more recent NBDPS control population. In a population-based, case-control study of childhood brain cancer that spanned cancer centers in eight states, Carozza et al. (1995) noted that 5% of control mothers took nitrosatable drugs anytime during pregnancy; medications taken included 24 different nitrosatable components.

Three subsequent case-control studies of childhood cancer used a broader classification scheme for nitrosatable drugs that was based on published experimental data or chemical structure (McKean-Cowdin et al.,^2003^; Cook et al.,^2004^; Cardy et al.,^2006^). Prevalence of any nitrosatable drug use among control mothers anytime during pregnancy ranged from 14.9% to 18.4%, but prevalence of use during the first trimester was not reported in these studies. Two of these studies also reported prevalences of nitrosatable intake by chemical group including 7.5 to 9% for secondary amines, 10 to 13% for tertiary amines, and 2.5 to 3% for amides (McKean-Cowdin et al.,^2003^; Cardy et al.,^2006^). We found the use of nitrosatable drugs in pregnancy among NBDPS participants substantially to be more common than in the study populations of these three studies, most likely because of a more comprehensive classification scheme based on a review by Brambilla and Martelli (2007). Another possible reason for the higher prevalence of nitrosatable drug use in the present study is that the index pregnancies of the NBDPS control mothers occurred more recently (1997–2005) than those of the cancer studies (index births occurred before 1995). Some types of drugs and patterns of use might have changed over time. In a study of over-the-counter medications taken during pregnancy, Werler et al. (2005) noted that use of dextromethorphan, diphenhydramine, and pseudoephedrine increased from 1976 to 2004. These drugs were among the most commonly taken nitrosatable drugs in the present study. Finally, interviews with the mothers in the childhood cancer studies predominantly took place more than 2 years after delivery of the index births and up to 20 years afterward, whereas NBDPS mothers were interviewed within 24 months after the expected due date of the index pregnancy. Results from several studies suggest that accuracy of recall may decrease as the span between pregnancy and maternal interview increases (Wilcox and Horney,^1984^; Koren et al.,^2004^).

Less research has been conducted on the relation between maternal nitrosatable drug use and adverse pregnancy outcomes besides childhood cancer. In several case-control studies of birth defects, nitrosatable drug use has ranged from 8.0% (Croen et al.,^2001^) and 8.7% (Brender et al.,^2004^) among control women during the periconceptional period (defined in both studies as 3 months preconception to 3 months postconception) to 10.7% anytime during pregnancy (Gardner et al.,^1998^).

Only one previously published study reported nitrosatable drug use by class of drug (Cardy et al.,^2006^). In the present study, NBDPS study participants took nitrosatable drugs that spanned a wide variety of indications and therapeutic actions. Examining nitrosatable drugs by indication and pharmacologic action might help to distinguish effects caused by nitrosatability from those caused by other pharmacologic effects or underlying illnesses.

This study provides some of the most recent data on the prevalence of nitrosatable drug exposure among women in childbearing years in which populations in all major regions of the United States are represented. The study also has several potential limitations. Prevalence estimates were based on women's reports of medications taken during pregnancy, and the drugs reported were not verified. Indeed, validating drug reports is a challenge because many over-the-counter and prescription drugs are not recorded in the medical records. In addition, although our classification of nitrosatable drugs is based on the most extensive published and up-to-date resources available, not all drugs have been tested. Brambilla and Martelli (2007) stated in their extensive review of nitrosatable drugs that “the number of theoretically nitrosatable drugs that have not been tested for the possible formation of genotoxic-carcinogenic N-nitroso derivatives is very high.”

In conclusion, nitrosatable drug use during the first trimester was common in this population of U.S. women in childbearing years. Many of the active ingredients identified as nitrosatable are available in over-the-counter medications. A substantial proportion of women who took these medications during the first trimester did not take supplements containing vitamin C on a daily basis until the third month of pregnancy. More research is needed regarding the effects of nitrosatable drug use on pregnancy outcomes in which the effects of such drugs are examined by their molecular structure, pharmacologic actions, and indication of use.
