# Supplementary material for: Prevalence and Patterns of Nitrosatable Drug Use among U.S. Women during Early Pregnancy
Source: Birth Defects Res A Clin Mol Teratol. 2011 Apr 6;91(4):258–64. doi: 10.1002/bdra.20808 (PMC3107676; doi:10.1002/bdra.20808)
Supplement: Supplementary file 1 [file bdra0091-0258-SD1.doc]

**APPENDIX**

| Drugs that have been determined to be nitrosatable by evaluation of their chemical structure, formation of a n-nitroso compound (NOC) by reaction with nitrite under standardized conditions, or genotoxic testing of nitrosation products with or without identification of NOC, reported by National Birth Defects Prevention Study control women, 1997-2005 | | | | |
| --- | --- | --- | --- | --- |
| **Drug** | **Compound type^a^** | **Drug Class** | **NOC** | **Ref** |
| Albuterol | 2 | Asthma, Beta adrenergic | Nitrosoalbuterol | b |
| Amitriptyline | 3 | Antidepressant, Tricyclic | NDMA | c |
| Amoxicillin | amide | Anti-infective, Beta lactam |  | b |
| Ampicillin | amide | Anti-infective, Beta lactam |  | b,c |
| Antipyrine | 3 | Analgesic | 4-Nitrosoantipyrine | c |
| Atenolol | 2, amide | Cardiovascular, Beta blocker | N-Nitrosoatenolol | c |
| Atropine | 3 | Anticholinergic | Nitroso-8-aza bicyclo octanol | b |
| Brompheniramine | 3 | Antihistamine | NDMA + others | b |
| Caffeine | 3, amide | Stimulant |  | b |
| Carbamazepine | 3, amide | Antiepileptic | N-Nitrosodibenzazepine, N-nitrosodihydrodibenzazepine | c |
| Carbinoxamine | 3 | Cough suppressant | NDMA | b |
| Cefaclor | amide | Anti-infective, Beta lactam |  | b |
| Cefalexin | amide | Anti-infective, Beta lactam |  | b,c |
| Chlorpheniramine | 3 | Antihistamine | NDMA | b,c |
| Chlorpromazine | 3 | Antiemetic, Phenothiazine | NDMA, N-nitrosodesmethylchlorpromazine | b |
| Cimetidine | 2, 3 | Gastrointestinal, H2 blocker | N-Nitrosocimetidine | b,c |
| Clemastine | 3 | Antihistamine |  | b |
| Clindamycin | 3, amide | Anti-infective, Macrolide |  | b |
| Clonidine | 2, 3 | Cardiovascular, Antihypertensive |  | c |
| Codeine | 3 | Analgesic, Opiod |  | b |
| Desipramine | 2, 3 | Antidepressant, Tricyclic | N-Nitrosodesipramine, N-nitrosodibenzazepine | c |
| Dexbrompheniramine | 3 | Antihistamine | NDMA + others | b |
| Dextromethorphan | 3 | Cough suppressant |  | b |
| Diazepam | 3, amide | Benzodiazepine |  | b,c |
| Dichloralphenazone | 3, amide | Migraine |  | b |
| Dicyclomine | 3 | Anticholinergic | NDEA | b |
| Diltiazem | 3, amide | Cardiovascular, Calcium channel blocker |  | c |
| Dimenhydrinate | 3, amide | Antiemetic, Antihistamine | NDMA + other | c |
| Diphenhydramine | 3 | Antihistamine | NDMA + other | b,c |
| Diphenoxylate | 3 | Antidiarrheal, Opioid | Nitrosopiperidine | b |
| Dipyrone | 3 | Analgesic | NDMA + other NOC | c |
| Doxycycline | 3, amide | Anti-infective, Tetracycline | NDMA | c |
| Doxylamine | 3 | Antihistamine | NDMA | b |
| Ephedrine | 2 | Decongestant | N-Nitrosoephedrine | c |
| Epinephrine | 2 | Asthma | Nitrosoepinephrine | c |
| Erythromycin | 3 | Anti-infective, Macrolide | NDMA | c |
| Fluoxetine | 2 | Antidepressant, SSRI |  | d |
| Furosemide | 2, amide | Cardiovascular, diuretic | Nitrosofurosemide | b,c |
| Hydralazine | 2, 3 | Cardiovascular, Antihypertensive |  | c |
| Hydrochlorothiazide | 2, amide | Cardiovascular, Thiazide | 4-Nitrosohydrochlorothiazide | b,c |
| Hydroxyzine | 3 | Antihistamine | N,N-Dinitrosopiperazine and other NOC | b,c |
| Hyoscamine | 3 | Anticholinergic | Nitroso aza bicyclo octanol | b |
| Isometheptane | 2 | Migraine | Nitrosoisometheptane | b |
| Lorazepam | amide | Benzodiazepine |  | b |
| Meclizine | 3 | Antihistamine | Dinitrosopiperazine + other | b |
| Meperidine | 3 | Analgesic, Opioid |  | b |
| Metformin | 2, 3 | Antidiabetic, Biguanide | NDMA | c |
| Methadone | 3 | Analgesic, Opioid | NDMA | c |
| Metoclopramide | 3, amide | Antiemetic, Prokinetic | NDEA + other | b,c |
| Metoprolol | 2 | Cardiovascular, Beta blocker | N-Nitrosometoprolol | c |
| Metronidazole | 3 | Anti-infective |  | b |
| Morphine | 3 | Analgesic, Opioid |  | b |
| Nicotine | 3 | Nicotine replacement | Nitrosonornicotine | c |
| Nifedipine | 2 | Cardiovascular, Calcium channel blocker |  | c |
| Nortriptyline | 2 | Antidepressant, Tricyclic |  | c |
| Oxycodone | 3 | Analgesic, Opioid |  | b |
| Paroxetine | 2 | Antidepressant, SSRI |  | d |
| Penicillin | amide | Anti-infective, Beta lactam |  | c |
| Phenobarbital | amide | Antiepileptic |  | b,c |
| Phenylephrine | 2 | Decongestant |  | b,c |
| Phenyltoloxamine | 3 | Antihistamine | NDMA | b |
| Phenytoin | amide | Antiepileptic |  | b,c |
| Prochlorperazine | 3 | Antiemetic, Phenothiazine | Dinitrosopiperazine | b,c |
| Promethazine | 3 | Antiemetic, Phenothiazine | NDMA + other | b,c,d |
| Propoxyphene | 3 | Analgesic, Opioid | NDMA + other | b |
| Propranolol | 2 | Cardiovascular, Beta blocker | N-Nitrosopropranolol | b,c |
| Pseudoephedrine | 2 | Decongestant |  | b,c |
| Pyrilamine | 3 | Antihistamine | NDMA | b |
| Ranitidine | 2, 3 | Gastrointestinal, H2 blocker | N-Nitroso-nitrolic acid derivative of ranitidine | b,c |
| Scopolamine | 3 | Anticholinergic |  | b |
| Sulfamethoxazole | amide | Anti-infective, Sulfonamide | Nitroso-sulfamethoxazole | b |
| Terbutaline | 2 | Asthma, Beta adrenergic |  | c |
| Tetracycline | 3, amide | Anti-infective, Tetracycline | NDMA | b,c |
| Timolol | 2, 3 | Cardiovascular, Beta blocker | N-Nitrosomorpholine | c |
| Tizanidine | 2 | Muscle relaxant |  | d |
| Triprolidine | 3 | Antihistamine | NPYR (N-Nitrosopyrrolidine) | b |
| Verapamil | 3 | Cardiovascular, Calcium channel blocker |  | c |
| Abbreviations: |  |  |  |  |
| Type of compound: | 2 =secondary amine, 3 =tertiary amine, amide=amide | | |  |
| N-nitroso compound: | NDEA: N-nitrosodiethylamine; NDMA: N-nitrosodimethylamine; NDPA: Nitrosodipropylamine; NPYR: Nitrosopyrrolidine | | |  |

^a^ References: Gillatt et al., 1984; Gillatt et al., 1985; McKean-Cowdin et al., 2003.

^b^Reference: McKean-Cowdin et al., 2003

^c^Reference: Brambilla and Martelli, 2007

^d^Reference: Ozhan and Alpertunga, 2003
